# An Evaluation of the Scholarly Activity Guidance and Evaluative (SAGE) Program

**DOI:** 10.15694/mep.2019.000065.1

**Published:** 2019-03-25

**Authors:** Emery Terrell, Amisha Agarwal, Timothy J. Wood, Hilary Writer, Catherine M. Pound

**Affiliations:** 1 University of Ottawa Faculty of Medicine; 2 Children’s Hospital of Eastern Ontario Research Institute; 3 Department of Innovation in Medical Education; 4 Department of Pediatrics

**Keywords:** research curriculum, residency program, program evaluation, resident scholar, research barriers

## Abstract

This article was migrated. The article was marked as recommended.

**Objective:** To evaluate the SAGE program five years following implementation.

**Methods:** Our program evaluation was based on Guskey’s five-level framework for evaluation of professional development. Residents and supervisors were invited to participate. Participants’ reactions, learning, perceived organization support, use of new knowledge or skills and learning outcomes were examined through questionnaires and interviews.

**Results:** 54% of residents and 65% supervisors were mostly or very satisfied with SAGE. 75% of residents felt there was moderate or great institutional support of resident research. Most residents and supervisors reported satisfaction with institutional research resources. Residents participating in the SAGE program reported a greater number of grant submissions and awards, but fewer conference presentations.

**Conclusions:** SAGE has been well received by residents and supervisors. Findings suggest the program has fostered the development of research abilities and improved institutional support. It remains unclear if demonstrable learning outcomes have increased since program implementation. We also identified several barriers which will serve as targets for program improvement in future.

## Introduction

Research training in residency is thought to improve clinical care through the development of critical appraisal and reasoning abilities, the promotion of evidence-based practice, and the fostering of lifelong learning skills ( Evans *et al.*, 1994, Abramson, 1977, Souba *et al.,* 1996, Pound *et al.,* 2015). As per the Royal College of Physicians and Surgeons of Canada (the Canadian medical specialty regulatory body), pediatric residents must participate in research activities during their training in order to be eligible to graduate from their residency program ( Royal College of Physicians and Surgeons of Canada, 2008).

Unfortunately, barriers to resident research are multiple, including inadequate protected time, lack of previous research training, insufficient financial support, vague curricular requirements, limited exposure to ongoing research projects ( Cull *et al.,* 2003), and a paucity of available and qualified mentors ( Neacy *et al.,* 2000, Cain *et al.,* 2001). While studies show that implementing protected time, research tracks, and/or research curricula result in increased scholarly output ( Stevenson *et al.,* 2017), there is rising concern that residency programs are failing to adequately promote and support research training for residents ( Cull *et al.,* 2003, Cabana *et al.,* 2014). Despite the importance of developing research skills in residents, there is a definite shortage of pediatric residency research training programs in Canada ( Royal College of Physicians and Surgeons of Canada, 2008), and a lack of information on the integration and effectiveness of these initiatives into existing residency programs ( Cull *et al.,* 2003, Roth, Chan and Vohra, 2006, Wood and Kronick, 2008, Ullrich *et al.,* 2003).

Additionally, a survey of pediatric residents and research supervisors conducted at our institution in 2012 showed that less than half of study participants felt that our center was supportive of resident research ( Moreau *et al.,* 2014). In response to the results of this survey, we implemented the Scholarly Activity Guidance and Evaluation (SAGE) program in September 2012, a program that focuses primarily on the development of residents’ research abilities as they relate to two main competencies ( Royal College of Physicians and Surgeons of Canada, 2008); the critical evaluation of information and its sources and the application of this information to clinical practice decisions, and the contribution to the creation, dissemination, application, and translation of new medical knowledge and practices.

The SAGE program includes two mandatory components. The first component consists of lectures and presentations on topics relevant to the development of scholarly projects (e.g. basic statistics, study designs, research ethics, formulating research questions, developing proposals and manuscripts) as well as mandatory journal clubs. The second component focuses on actively guiding and supporting residents through the development and implementation of their scholarly project, to maximize success. In brief, residents choose a research supervisor and work under their supervision for the duration of their project. Each resident’s progress is reviewed at least twice yearly by a committee of eight individuals with expertise in various aspects of research (e.g. methodology, bioethics, grantsmanship, medical education) and written feedback is provided to both the resident and the supervisor. Residents are eligible for up to 12 weeks of protected research time and have access to internal funding for their projects. In addition, all residents are expected to present at SAGE rounds, where projects at any stage of development are discussed with hospital staff and research experts. The SAGE committee chair, a pediatrician with research expertise, meets with each resident individually at the beginning of residency training, and again as needed or requested by individual residents if difficulties arise (Information on the SAGE committee resources can be found in Supplementary Material).

Five years after inception of the SAGE program, we wished to evaluate its success with respect to resident and supervisor satisfaction, residents self-assessed knowledge, impact of the program on resident research culture at our institution, program utilization, and resident research productivity.

## Methods

We based our evaluation of the SAGE program on Guskey’s five-level framework for the evaluation of professional development ( Guskey, 2000), and examined five levels of information: participants’ reactions, participants’ perceived learning, organization support, participants’ use of new knowledge or skills, and participants’ learning outcomes. Applied to SAGE, the following questions guided our program evaluation:

1. Are key stakeholders (i.e., residents, research supervisors) satisfied with the SAGE program?
2. Do residents perceive that their knowledge of specific research topics and processes has improved because of SAGE?
3. How has SAGE enhanced the resident research culture?
4. Are residents using the resources and feedback provided by SAGE?
5. How has SAGE contributed to resident research productivity?

Ethics approval was obtained by the institutional Research Ethics Board for all study components. To ensure that the questionnaires distributed to study participants were appropriate, relevant and not missing any pertinent items, a set of reviewers who have knowledge of SAGE reviewed the questionnaires for content evidence.

### Participants’ Reactions and Learning

All residents and resident research supervisors who participated in the SAGE program between July 1 2016 and June 30 2017 were invited to complete an online questionnaire. Survey questions were developed based on a modified version of the Client Satisfaction Questionnaire ( Larsen *et al.,* 1979), which uses a 4-point rating scale ranging from positive to negative reactions. Two questions in the resident and supervisor questionnaires were based on the results of a previous needs assessment ( Moreau *et al.,* 2014). One question in the resident questionnaire focused on self-reported learning.

### Organization Support & Participants’ Use of New Knowledge or Skills

One question in the resident and supervisor questionnaires focused on perceived organization support of resident research. Counts of numbers of residents using their dedicated research blocks and the number of residents accessing CHEO’s clinical research unit services were obtained retrospectively. Additionally, previously or currently involved residents and supervisors were invited by electronic information letter to participate in interviews with a research assistant to explore their experiences, reactions and thoughts with respect to the SAGE program and the institutional resident research culture.

### Participants’ Learning Outcomes

A second survey was sent to past residents in order to compare learning outcomes for trainees who graduated prior to SAGE implementation (prior to September 2012) to those who were exposed to SAGE: counts were obtained on the number of (a) both internal and external grant applications submitted by residents, (b) successful resident research grants (both external and internal) obtained, (c) residents presenting their research findings at local, national, or international conferences, (d) residents who submitted their research to peer-reviewed journals, and (e) residents whose research was accepted for publication in peer-reviewed journals. A PubMed database search was conducted to determine number of published articles by residents in both pre- and post-SAGE groups during their general paediatrics training at CHEO. This observation period included an additional 2 years following the end of the resident’s training at CHEO to allow for publication delay related to preparation of a manuscript, peer-review process, and publication.

### Data collection

Survey responses and some learning outcome data were collected and managed using REDCap (Research Electronic Data Capture) electronic data capture tools hosted and supported by the CHEO Research Institute. REDCap is a secure, web-based application designed to support data capture for research studies ( Harris *et al.,* 2009). Data with regards to resource utilization was collected through the Clinical Research Unit (CRU) and Postgraduate Medical Education Departments at CHEO. Resident publications were compiled by PubMed search conducted by one of the authors (ET).

### Quantitative analysis

Resident and supervisor questionnaire responses (participants’ reaction & learning) were summarized using descriptive statistics (frequency and percent). Additionally, participant learning outcomes were summarized using frequency and percent, between residents in the pre- and post-SAGE program implementation groups. The Fisher’s Exact test was used to statistically compare all categorical learning outcomes. Learning outcomes based on counts were not statistically compared as individual exposure time was unknown. Two-sided p-values less than 0.05 were considered statistically significant. All statistical analyses were performed using R statistical software version 3.4.2 1 ( R Core Team, Vienna, Austria, 2017).

### Qualitative analysis

Due to the very small number of participants recruited for interviews, a formal rigorous qualitative analysis could not be performed. Audio-recordings were transcribed verbatim and themes were identified and summarized by the author who conducted the interviews (ET). The author reviewed each transcript, annotated phrases within the text, and identified recurring and significant ideas. These ideas were coded and cross-referenced with the other transcripts: common themes were described and organized into overarching themes.

## Results/Analysis

### Participants’ Reactions and Learning

Online questionnaires were sent to eligible participants, 38 residents and 32 supervisors. Twenty-four residents and 20 resident supervisors responded to the online questionnaires, with respective response rates of 63.2% and 62.5%. In the resident group, 1 (4.2%) was in first year, 11 (45.8%) were in second year, 6 (25.0%) were in third year and 6 (25.5%) were in fourth year. Fifty-four per cent of the responding residents were overall mostly or very satisfied with the SAGE program, 38% were indifferent or mildly dissatisfied, and 8% were quite dissatisfied. Also, 83% of participating residents would come back to the SAGE program to seek research guidance again. Fifty per cent of participants felt that their needs had mostly, or all been met, 50% felt a few of their needs had been met, and no residents felt that none of their needs were met. Most of the participating supervisors had supervised 1 to 2 residents in the past year. One had supervised 3 to 5, and one did not supervise anyone. Sixty-five per cent of the responding supervisors were mostly or very satisfied with the guidance their residents received from the SAGE program, while 35% were mildly dissatisfied or indifferent. Seventy-nine per cent felt that most or all the residents’ needs had been met by the SAGE program, and 95% would recommend that their residents seek guidance again from the program. Table 1 summarizes residents’ self-assessed learning in relation to the SAGE program.

**Table 1.** Resident survey responses (N = 24)

Level of comfort with...	Not at all comfortable (% of participants)	Somewhat comfortable (% of participants)	Comfortable (% of participants)	Very comfortable (% of participants)	Don’t know (% of participants)
Ability to generate research idea?	0.0	45.8	45.8	8.3	0.0
Ability to formulate research questions?	4.3	56.5	30.4	8.7	0.0
Ability to perform a literature search?	4.2	33.3	50.0	12.5	0.0
Ability to critically evaluate research literature in your area of interest?	4.2	37.5	45.8	8.3	4.2
Ability to prepare a research proposal?	12.5	45.8	29.2	8.3	4.2
Ability to prepare research budgets?	54.2	37.5	8.3	0.0	0.0
Knowledge of research funding application process?	41.7	37.5	20.8	0.0	0.0
Knowledge of ethical consideration when conducting research with children and families?	16.7	41.7	33.3	8.3	0.0
Knowledge of the research ethics application process?	16.7	37.5	25.0	20.8	0.0
Ability to collect quantitative data?	12.5	33.3	41.7	8.3	4.2
Ability to collect qualitative data?	8.3	54.2	29.2	4.2	4.2
Ability to analyze and interpret quantitative data?	25.0	54.2	16.7	0.0	4.2
Ability to analyze and interpret qualitative data?	29.2	54.2	12.5	0.0	4.2
Ability to present research findings?	4.2	41.7	33.3	16.7	4.2
Ability to write a manuscript for a journal?	4.3	52.2	17.4	17.4	8.7

### Organization Support & Participants’ Use of New Knowledge or Skills

At the organizational level, 75% and 80% of residents and supervisors, respectively felt that CHEO was moderately or greatly supportive of resident research, 21% and 15% reported it was supportive to a small extent and 4% of residents felt the institution was not supportive; 5% of supervisors did not know. The majority of survey respondents reported accessing institutional research supports including the clinical research unit, librarian, SAGE program and SAGE rounds. The access of these resources reported by residents and supervisors, as well as satisfaction with available resources and perceived institutional recognition are summarized in tables 2 and 3 respectively.

**Table 2.** Access to Research Resources (Residents N = 24, Supervisors N = 20)

Research Resource	Reported Access (%)	
	Resident	Supervisors’ impressions of residents accessing research resources
Clinical Research Unit	58.3	80.0
Librarian	75.0	70.0
SAGE program	79.2	80.0
Attended SAGE rounds on 1-3 occasions	79.2	N/A ^1^
Presented at SAGE rounds	62.5	N/A ^1^

^1^ *SAGE rounds questions were included in the resident questionnaire only. Supervisors were not asked about SAGE rounds attendance or satisfaction. Description of SAGE rounds available in Appendix 1.*

**Table 3.** Satisfaction with Research Resources (Residents N = 24, Supervisors N = 20)

To what extent does ____ meet your/your resident’s research needs or fulfill your/your resident’s expectations?	Not at all (%)	To a small extent (%)	To a moderate extent (%)	To a great extent (%)	Don’t know (%)
Journal club					
Resident	16.7	29.2	37.5	8.3	8.3
Supervisor	0.0	15.0	0.0	0.0	85.0
SAGE Rounds					
Resident	4.2	33.3	33.3	12.5	16.7
Supervisor	0.0	10.0	45.0	25.0	20.0
Resident Research Day					
Resident	0.0	0.0	45.8	37.5	16.7
Supervisor	0.0	10.0	25.0	40.0	25.0
Feedback from SAGE committee					
Resident	4.2	41.7	45.8	8.3	0.0
Supervisor	10.0	15.0	30.0	35.0	10.0
Extent to which resident research accomplishments are recognized?					
Resident	4.2	20.8	25.0	8.3	41.7
Supervisor	0.0	5.0	35.0	25.0	35.0

Since 2016, 35 of 43 residents have accessed their dedicated research blocks with an average of 24 days used (median = 14, IQR = 37). Comparison with the pre-SAGE implementation could not be performed as this data was not collected prior to 2016. Residents used an average of 4 additional protected days in 2017-2018 (median = 2, IQR = 9) as compared to the previous year. Also, 33 residents submitted requests to access our institutions’ clinical research unit in 2016 as compared to 62 residents in 2017.

Multiple invitations to participate in the interviews were sent to eligible residents and supervisors. Four residents and 3 supervisors agreed to participate in semi-structured interviews. Three main themes emerged from transcript analysis: positive feedback, constructive criticism and conflicting views ( table 4).

**Table 4.** Emerging Themes from Resident and Supervisor Interviews

**Areas of strength** Formalized expectations with more explicit objectives and goals. Increased program structure with a council of mentors. Perceived increased in scholarly work produced. Provided and connected trainees with various research resources.
**Areas for improvement** Definition of scholarly project remains poorly understood. Concern that increased emphasis on scholarly activity has resulted in narrower scope of projects and decreased incentive for creativity so that residents may produce and publish. Introduction of more administrative barriers. More research methodology teaching and mentorship still needed. Importance of adequate support and mentorship.
**Conflicting views** Shift in responsibility and accountability between residents and supervisors since implementation. Committee feedback perceived to be more useful by supervisors compared to residents. Organization of program and committee.

### Participants’ Learning Outcomes

A total of 8 of the 30 residents approached (26.7% response rate) who graduated prior to 2012, and 37 of the 40 residents (92.5% response rate) who graduated after 2012 participated in the outcome questionnaire. Responses are summarized in Table 5. A PubMed database search performed to confirm resident publications showed an average number of published articles of 0.72 before SAGE implementation and 0.73 after (median 1 in both groups).

**Table 5.** Learning outcomes in pre-SAGE (N = 8) and post-SAGE (N = 37) resident graduates

	Pre-SAGE (N=8)		Post-SAGE (N=37)		
Learning outcome	n/N _available_ (%)	N (%)	n/N _available_ (%) ^1^	N (%)	p-value
Applied for grants to fund your study/scholarly project while a resident at CHEO	8 /8 (100.0)	2 (25.0)	37 /37 (100.0)	20 (54.1)	0.24
Number of internal grant applications submitted	1 /2 (50.0)		20 /20 (100.0)		1.00
1		1 (100.0)		17 (85.0)	
2		0 (0.0)		3 (15.0)	
Number of external grant applications submitted	1 /2 (50.0)		2 /20 (10.0)		1.00
1		1 (100.0)		1 (50.0)	
2		0 (0.0)		0 (0.0)	
3		0 (0.0)		0 (0.0)	
4		0 (0.0)		1 (50.0)	
Awarded grants to fund your study/scholarly project	8 /8 (100.0)	2 (25.0)	37 /37 (100.0)	14 (37.8)	0.69
Number of internal grants awarded	1 /2 (50.0)		14 /14 (100.0)		1.00
1		1 (100.0)		12 (85.7)	
2		0 (0.0)		2 (14.3)	
Number of external grants awarded	1 /2 (50.0)		0 /14 (0.0)		1.00
1		1 (100.0)		0 (0.0)	
Presented research findings at conference	8 /8 (100.0)	6 (75.0)	37 /37 (100.0)	19 (51.4)	0.27
Total number of local presentations	3 /6 (50.0)	N=3	14 /19 (73.7)	N=17	NA ^1^
Total number of national presentations	5 /6 (83.3)	N=4	13 /19 (68.4)	N=10	NA ^1^
Total number of international presentations	4 /6 (66.7)	N=3	15 /19 (78.9)	N=11	NA ^1^
Submitted research findings to peer-reviewed journal	8 /8 (100.0)	4 (50.0)	36 /37 (97.3)	17 (47.2)	1.00
Number of articles submitted	4 /4 (100.0)	N=6	17 /17 (100.0)	N=19	NA ^1^
Number of articles accepted	4 /4 (100.0)	N=5	15 /17 (88.2)	N=11	NA ^1^

^1^ Cannot compute due to unknown individual exposure period.

## Discussion

In this study, we show that most residents and supervisors are satisfied or mostly satisfied with our SAGE program. Participating in the SAGE program was also associated with the majority of residents feeling moderately comfortable or comfortable with scholarly competencies. Residents and supervisors’ perception of institutional resident research support has improved, and there is an increase in the use of learning resources and supports.

Residents’ views of the SAGE program were moderately positive, and residents reported a certain level of comfort with various fundamental research skills. This is in keeping with current literature showing that dedicated research programs can improve knowledge, skills, learning satisfaction and measurable learning outcomes ( Kanna *et al.,* 2006, Jain *et al.,* 2016). Our survey also identified areas of knowledge gaps, specifically preparing research budgets, developing funding applications as well as interpretation of quantitative and qualitative analyses, suggesting areas for future program improvement. Interestingly, similar gaps in knowledge have also been identified in a critical care resident research needs assessment ( Jain *et al.,* 2016), which used data from institutions across Canada including our own, suggesting that these gaps may extend across specialty training programs and are not specific to paediatrics at our institution ( Jain *et al*., 2016).

Although we were unable to perform a formal analysis due to sample size, the interviews with residents and supervisors were largely in keeping with the quantitative data: both groups identified a defined program structure, access to various research resources, increased research mentorship and increased scholarly work as benefits of the SAGE program. However, the interviews highlighted several issues where stakeholders had conflicting views.

Some supervisors felt that SAGE had increased their accountability in supervising resident projects, while others felt that it had taken away some of their responsibilities by having the SAGE program provide a timeline and ensure that residents stay on track. Supervisors generally perceived program timelines, support and general organization as adequate while residents felt these areas could be improved with the addition of more research mentors. In addition, supervisors were more satisfied by the feedback provided by the SAGE committee as compared to the residents, who voiced not always finding the feedback helpful.

The interviews also helped explore areas for improvement that were identified by both residents and supervisors in the online surveys. One such issue was the definition and scope of resident scholarly projects: both residents and supervisors reported having difficulty creating a research question with realistic scope for completing during residency while still producing worthwhile research. This is a barrier that has already been described in previous studies assessing residency research projects ( Levine, Hebert and Wright, 2005). Residents also voiced feeling increased pressure to produce a project within a timeline while balancing clinical duties, as described in the literature ( Yuan, Xu and Hu, 2013). The other area for improvement raised by residents and supervisors was to increase methodology training and research mentorship earlier on in residency. Based on this feedback, a one-week research course has now been introduced for first year residents upon entering our training program.

Residents and supervisors’ perceptions of institutional support of resident research increased considerably post SAGE implementation. A survey distributed at our institution in 2011 showed that 32% of residents and 26% of supervisors perceived our institution to be supportive of resident research ( Moreau *et al.,* 2014), while in our current study, 75% of residents and 80% of supervisors feel that CHEO is moderately or greatly supportive of resident research. This suggests that the implementation of the SAGE program has had a measurable positive impact at an institutional level. This may be due to a greater number of resident research activities, journal club, SAGE rounds and committee feedback, raising awareness of the increased importance of scholarly activities for residents. Interestingly, supervisors tended to rate the SAGE resources more positively than residents did and were less divided in terms of perceived institutional recognition of resident research accomplishments. While the reasons for this remain unclear, this may be because residents were the ones to access resources and therefore more likely to experience day-to-day organizational challenges, while supervisors had a more removed role and observed the results of their residents’ accessing SAGE resources.

The number of residents using their dedicated research blocks was not recorded prior to 2016; however, since 2016, there has been a trend of increasing numbers of residents accessing protected research time and increasing number of days used. Of interest, the senior residents had a greater increase in the number of days used compared to their junior resident colleagues. Along with this increase in research block usage, there has been an increase in the number of residents consulting the clinical research unit and the number of submitted consult requests. Although speculation, this could be the result of increased perceived institutional support and recognition of resident research since the implementation of the SAGE program: that residents are more aware of these resources and feel comfortable accessing them. It will be interesting in the future to examine whether this trend continues.

The participants’ outcome online survey was completed by residents who completed their training either pre-SAGE or post-SAGE implementation with the majority of responses from the post-SAGE group. There was no significant difference in the number of grant applications, grants awarded to residents, and conference presentations reported between the pre and post-SAGE implementation periods, which may be due to the small number of recruited residents. The PubMed search of publications also showed no difference between the two groups; however, it is important to note that the PubMed observation period was extended 2-years post-graduation to allow for delays in publication with the intent of capturing the publication of scholarly projects from the resident’s general pediatrics training at our institution. For some of the residents in the post-SAGE group, this observation period would end in 2019 and thus it is possible that the final number of publications from the group exposed to SAGE could increase and surpass the pre-SAGE group. This would align with previous studies showing improved learning outcomes following the implementation of residency research programs ( Basu *et al.,* 2012).

### Study Limitations

This study had several limitations. The sample size was small, due to a relatively small number of residents in our training program. However, the response rates for the quantitative portion of the study were solid for a single-centre study, with the exception of the participants’ learning outcome survey for graduates before 2012 (27%), as they were mostly well above the 30% threshold previously identified in the literature as being typical for organization surveys (Church and Waclawski, 1998) and our study population surveyed ( Asch *et al.,* 2000).

Although the response rate was solid for the questionnaire part of the study (except for the pre-SAGE learning outcome questionnaire group), the number of participants who participated in the semi-focused interviews, despite multiple attempts at recruitment, was quite restricted, limiting interpretation and analysis of the qualitative data. In addition, the comparison of resident outcomes between those in the pre-SAGE group versus the post-SAGE group was limited by a small sample size, therefore resulting in low statistical power. We were also unable to compare presentation and publication rates before and after implementation as individual follow-up time was unknown.

## Conclusion

Since its implementation in 2012, the SAGE program has been largely well received by residents and supervisors, and perception of institutional support for resident research has considerably improved. Our findings suggest the program has been associated with development of research abilities and improved institutional support and recognition. It remains too early to determine whether learning outcomes, such as number of publications, have increased following the introduction of the SAGE program but there was a trend towards higher number of awarded grants post SAGE. This study identified and addressed several barriers and gaps in knowledge, all previously described in the literature, which will serve as targets for further program improvement in the future. This SAGE program could also serve as a guide for the development of similar programs at other institutions to promote postgraduate medical research in such a way that addresses known barriers, seeks and incorporates ongoing feedback from stakeholders, and may improve quantifiable learning outcomes.

Abbreviations

Children’s Hospital of Eastern Ontario (CHEO), Scholarly Activity Guidance and Evaluative (SAGE) Program, Research Electronic Data Capture (REDCap)

## Take Home Messages

• Dedicated residency research programs, such as SAGE, can improve knowledge, skills, learning satisfaction and measurable learning outcomes
• The implementation of SAGE was wellreceived by residents and supervisors, and has had a measurable positive impact at an institutional level
• SAGE helped identify current gaps in residency research training which can serve as areas for future program improvement
• SAGE may serve as a guide for the development of similar programs at other institutions to promote postgraduate medical research in such a way that addresses known barriers, seeks and incorporates ongoing feedback from stakeholders, and may improve quantifiable learning outcomes.

## Notes On Contributors

Emery G. Terrell, BSc, is an MD candidate at the University of Ottawa in Ottawa, Canada.

Amisha Agarwal, MSc, is a methodologist with the Clinical Research Unit at the Children’s Hospital of Eastern Ontario Research Institute, Ottawa, Canada.

Timothy J. Wood, PhD, is a Professor in the Department of Innovation in Medical Education at the University of Ottawa, Ottawa, Canada.

Hilary Writer, MD, is an Assistant Professor, and was the Postgraduate Medical Education Program Director for the pediatrics residency program at the University of Ottawa until December 2018, Canada.

Catherine Pound, MD, is an Associate Professor at the University of Ottawa, and the Consulting Pediatrics Division Research Director at the Children’s Hospital of Eastern Ontario, Ottawa, Canada. She was also the founder and chair of the SAGE program until June 2018.

## Appendices

### Appendix

**Appendix 1. SAGE Resources**

***SAGE Timeline.*** Each resident enrolled in the general paediatrics residency training program must participate in the SAGE program for the duration of their pediatric residency. In the first two years of residency, residents are expected to identify a research idea, question(s), project supervisor and to draft a research proposal. In the remaining years, residents carry out their proposed research by meeting with content and methodological experts, submitting their projects to the required ethics board(s), applying for research funding, collecting and analyzing their data, writing a draft manuscript and disseminating their findings at academic conference.

***SAGE Handbook.*** A key resource, this handbook describes the SAGE program including yearly expectations and timelines.

***SAGE Rounds***. Lunchtime rounds which occur four times a year. These rounds are a forum with informal presentations of research-in-progress by 2 to 3 residents to other residents, supervisors and staff with research expertise who provide immediate constructive feedback. Depending on the stage of the projects, these rounds can be used as an opportunity to bounce ideas off the audience or discuss challenges that residents are experiencing.

***SAGE Committee***. Provides residents with consistent guidance and mentorship. This committee, which is chaired by a Scholarly Activity Coordinator, consists of 10 to 12 individuals and includes representatives from the primary stakeholder groups in resident research. Residents must submit a research progress report to this committee every 6 months outlining the current state of their required scholarly projects and any challenges they are facing. The committee reviews the reports, provides written feedback to each resident, connects residents with content and methodological experts, and reviews their draft ethics and funding applications prior to submission for detailed feedback.

***SAGE Resident Assessment.*** A closed and open-ended assessment form to provide formative feedback to each resident. The committee also judges the scientific score and the relevance score of the proposal. If the committee has major concerns with a resident’s progress, the Scholarly Activity Coordinator meets with the resident to discuss challenges and brainstorm solutions. Lastly, the Scholarly Activity Coordinator, in consultation with the other members of the SAGE committee, determines if residents have met the program’s scholarly expectation at graduation. To successfully meet the program’s expectations, residents must meet one of the following: (a) presentation of their project at the Institution’s Annual Resident Research Day, (b) presentation of the project at a local, provincial, national, or international meeting, or (c) submission of a manuscript to a peer-reviewed journal.
