# Supplementary material for: An Evaluation of the Scholarly Activity Guidance and Evaluative (SAGE) Program
Source: MedEdPublish (2016). 2019 Mar 25;8:65. [Version 1] doi: 10.15694/mep.2019.000065.1 (PMC10712647; doi:10.15694/mep.2019.000065.1)
Supplement: Peer review history

# Reviewer report: Reviewer response for version 1

**DOI:** 10.21956/mep.19782.r29733

**Published:** 2019-04-03

**Authors:** Balakrishnan Nair

**Affiliations:** 1 Centre for Medical Professional Development and University of Newcastle

This review has been migrated. The reviewer awarded 4 stars out of 5 Research training should be part of medical education, particularly at post graduate level. This will help the trainees to do research , critically appraise literature and help them to translate evidence into practice. This study has reviewed the impact of research training amongst post graduate trainees and is well conducted. As the authors state the sample size was small. Conclusions are appropriate . It will be good to get some longitudinal outcome of this cohort

Reviewer Expertise:

NA

No decision status is available

# Reviewer report: Reviewer response for version 1

**DOI:** 10.21956/mep.19782.r29732

**Published:** 2019-03-26

**Authors:** Sateesh Babu Arja

**Affiliations:** 1 Avalon University School of Medicine

This review has been migrated. The reviewer awarded 4 stars out of 5 I thoroughly enjoyed reading this article. It is a very well written paper. The authors clearly explained their SAGE program and how it is implemented. All medical schools' curricula these days are teaching research methodology and principles if not a mandatory research project. It is good to see residents continuing and employing the knowledge that they have learned in clinical research during their residency program. It is interesting to see this basic research methodology and principles are brushed up again in the SAGE program. The only comment from my side is instead of using the scale like not at all satisfied, to a small extent, moderate extent, great extent and don't know, I would have used very dissatisfied, dissatisfied, no opinion, satisfied, very satisfied. This would have given a better interpretation. My comment is the same for table one. This paper is good for faculty members and supervisors involved in the implementation of research projects for medical students and residents.

Reviewer Expertise:

NA

No decision status is available
